# Genome-Wide Identification of Antimicrobial Intrinsic Resistance Determinants in *Staphylococcus aureus*

**DOI:** 10.3389/fmicb.2016.02018

**Published:** 2016-12-19

**Authors:** Martin Vestergaard, Bingfeng Leng, Jakob Haaber, Martin S. Bojer, Christina S. Vegge, Hanne Ingmer

**Affiliations:** Department of Veterinary Disease Biology, Faculty of Health and Medical Sciences, University of CopenhagenFrederiksberg, Denmark

**Keywords:** *Staphylococcus aureus*, antimicrobial agents, intrinsic resistance, potentiator targets, *Galleria mellonella*

## Abstract

The emergence of antimicrobial resistance severely threatens our ability to treat bacterial infections. While acquired resistance has received considerable attention, relatively little is known of intrinsic resistance that allows bacteria to naturally withstand antimicrobials. Gene products that confer intrinsic resistance to antimicrobial agents may be explored for alternative antimicrobial therapies, by potentiating the efficacy of existing antimicrobials. In this study, we identified the intrinsic resistome to a broad spectrum of antimicrobials in the human pathogen, *Staphylococcus aureus*. We screened the Nebraska Transposon Mutant Library of 1920 single-gene inactivations in *S. aureus* strain JE2, for increased susceptibility to the anti-staphylococcal antimicrobials (ciprofloxacin, oxacillin, linezolid, fosfomycin, daptomycin, mupirocin, vancomycin, and gentamicin). Sixty-eight mutants were confirmed by *E*-test to display at least twofold increased susceptibility to one or more antimicrobial agents. The majority of the identified genes have not previously been associated with antimicrobial susceptibility in *S. aureus*. For example, inactivation of genes encoding for subunits of the ATP synthase, *atpA*, *atpB*, *atpG* and *atpH*, reduced the minimum inhibitory concentration (MIC) of gentamicin 16-fold. To elucidate the potential of the screen, we examined treatment efficacy in the *Galleria mellonella* infection model. Gentamicin efficacy was significantly improved, when treating larvae infected with the *atpA* mutant compared to wild type cells with gentamicin at a clinically relevant concentration. Our results demonstrate that many gene products contribute to the intrinsic antimicrobial resistance of *S. aureus*. Knowledge of these intrinsic resistance determinants provides alternative targets for compounds that may potentiate the efficacy of existing antimicrobial agents against this important pathogen.

## Introduction

Antibiotic resistant bacteria are a growing global issue that reduces our ability to cure bacterial infections (Gould, 2009; Davies and Davies, 2010). The limited success in identifying promising new antimicrobial entities led to a search for new approaches to counter the increasing problems of antibiotic resistance (Butler et al., 2013). One approach suggested is to re-sensitize resistant bacteria to an antimicrobial agent by potentiating the efficacy of an antimicrobial with a helper-drug. The helper-drug can target gene products that by any mechanism aid bacteria to resist higher concentrations of an antimicrobial (Pieren and Tigges, 2012). Besides the ability of bacteria to acquire antimicrobial resistance *via* horizontal gene transfer or spontaneous mutations, they can also be intrinsically resistant to antimicrobials (Cox and Wright, 2013). Intrinsic resistance to antimicrobials has traditionally been attributed to reduced permeability of the cell envelope, presence of inactivating enzymes or efflux pumps that can extrude the antimicrobial agents (Cox and Wright, 2013). Clinical use of potentiators have been applied successfully to the antimicrobial class of β-lactams, where β-lactamase inhibitors can significantly enhance the efficacy of β-lactams (Drawz and Bonomo, 2010). An analogous approach has been pursued by limiting the active efflux of antimicrobial agents by efflux pump inhibitors (Lomovskaya and Bostian, 2006), which have been shown to potentiate the efficacy of, e.g., levofloxacin in *Pseudomonas aeruginosa* (Renau et al., 1999) and norfloxacin in *Staphylococcus aureus* (Stermitz et al., 2000). However, efflux pumps inhibitors have not yet been approved for treatment of human infections due to tolerability issues (Fernebro, 2011).

It has recently been established from genome-wide studies of intrinsic resistance determinants in the Gram-negative bacteria *Escherichia coli* (Tamae et al., 2008; Liu et al., 2010), *Acinetobacter baylyi* (Gomez and Neyfakh, 2006) and *Pseudomonas aeruginosa* (Fajardo et al., 2008; Dötsch et al., 2009; Alvarez-Ortega et al., 2010; Gallagher et al., 2011; Krahn et al., 2012) that large and complex networks of both established and yet uncharacterized gene products contribute to reduce the inhibitory activity of antimicrobial agents. Equivalent comprehensive genome-wide studies of intrinsic resistance determinants in Gram-positive bacteria have not been performed, except for a single study that determined the intrinsic resistance of *S. aureus* to vancomycin, nisin and daptomycin (Blake and O’Neill, 2013). *Staphylococcus aureus* is an opportunistic pathogen with the capability to cause a wide range of diseases, ranging from systemic to skin infections (Lowy, 1998). The ability to treat *S. aureus* infections has been greatly hampered by the ability of this pathogen to develop resistance to antimicrobials (Sakoulas and Moellering, 2008; Chambers and DeLeo, 2009), which necessitates an understanding of determinants that contribute to the reduced susceptibility of *S. aureus* to antimicrobial agents.

In the present study, we identified genetic determinants contributing to the intrinsic resistance of *S. aureus* to eight different antimicrobials (ciprofloxacin, oxacillin, linezolid, fosfomycin, daptomycin, mupirocin, vancomycin, and gentamicin). We employed the Nebraska Transposon Mutant Library of 1920 single-gene inactivations in *S. aureus* JE2 (Fey et al., 2013) to screen for mutants that were unable to grow at sub-inhibitory concentrations of the antimicrobials. We identified multiple genes not previously recognized as modulators of antibacterial sensitivity, thus providing novel targets for the development of antibacterial sensitizer compounds.

## Materials and Methods

### Bacterial Strains, Growth Conditions and Chemicals

The strains used in the study include *S. aureus* JE2 (plasmid-cured derivative of USA300 LAC) and all derivative strains within the Nebraska Transposon Mutant Library (NTML), consisting of 1920 unique transposon mutants with inactivation of non-essential genes (Fey et al., 2013). The *bursa aurealis* transposon used to create the collection contains the resistance cassette *ermB* conferring resistance to erythromycin (Fey et al., 2013). All bacterial strains were cultured at 37°C in tryptic soy broth (TSB) or on tryptic soy agar (TSA), with antimicrobial agents added as indicated. Antimicrobial agents used in the study were erythromycin (Sigma), ciprofloxacin (Sigma), oxacillin (Sigma), linezolid (Sigma), fosfomycin (Sigma), daptomycin (Santa Cruz Biotechnology), mupirocin (Sigma), vancomycin (Sigma) and gentamicin (Sigma). Transduction of *atpA*::ΦNΣ into wild type (WT) JE2 was performed with bacteriophage φ11, by selecting for transductants on erythromycin plates (5 μg/ml) (Fey et al., 2013).

### Screening for Increased Antimicrobial Susceptibility

To screen for increased susceptibility among the NTML strains, we first determined the minimum inhibitory concentration (MIC) of WT strain JE2 to ciprofloxacin, oxacillin, linezolid, fosfomycin, daptomycin, mupirocin, vancomycin, and gentamicin. MIC was determined using a twofold microbroth dilution assay according to Clinical and Laboratory Standards Institute (CLSI) guidelines (CLSI, 2009), except that cation-adjusted Mueller Hinton broth was substituted with TSB.

The NTML is stored in glycerol at -80°C in 20 96-well microtiter plates. Material from the frozen stock was transferred directly with a Deutz 96 cryoreplicator (Duetz et al., 2000) from the 96-wells microtiter plates onto TSA plates supplemented with 5 μg/ml erythromycin (to prevent the growth of contaminants during the screen, as all the strains in the NTML are resistant to erythromycin; Fey et al., 2013) and 0.5x MIC of the respective antimicrobial agent. The plates were incubated at 37°C for 24 h and visually inspected for lack of growth of individual mutants.

### Determination of MIC of Hypersusceptible Mutants

The MIC was determined for all strains displaying growth deficiency in the hypersusceptibility screen to the respective antimicrobial agent and WT strain JE2 using *E*-tests (bioMérieux) performed on TSA plates. No erythromycin was supplemented to the plates during MIC determination with *E*-tests. The MIC was determined upon incubation at 37°C for 22 h and interpreted according to the guidelines of the manufacturer.

### Treatment of *Galleria mellonella* With Gentamicin

To investigate if increased antimicrobial susceptibility could be detected *in vivo*, we employed the *Galleria mellonella* infection model (Desbois and Coote, 2011; Ramarao et al., 2012). Healthy 5th-instar wax moth larvae weighting approximately 250 mg were randomly picked from a batch purchased at a local pet store and divided into six groups with 20 larvae in each. Virulence of *S. aureus* WT and the *atpA* mutant were compared by injecting 20 larvae with 10 μl (containing 1 × 10^7^ CFU) with a Hamilton syringe into the hemocoel through the lowest left proleg. Survival of *G. mellonella* was monitored for 120 h. For treatment efficacy of gentamicin against *S. aureus*, 20 larvae for each group were injected with 1 × 10^7^ CFU of WT or the *atpA* mutant. One hour post infection, the larvae were injected with 10 μl gentamicin (1 mg/kg bodyweight) in the lowest right proleg. Gentamicin therapy was repeated every 24 h for a total of 72 h and survival of *G. mellonella* was monitored for 120 h. A control group for toxicity of gentamicin (1 mg/kg bodyweight) as well as for non-treated (inoculated with phosphate-buffered saline) were included. The data was analyzed in GraphPad Prism 4 (GraphPad Software Inc.) using the Kaplan–Meier method and statistical difference determined using log rank test.

### Chromosomal Reconstruction of the *atpA* Mutant

Chromosomal reconstruction of the *atpA* mutant was achieved by use of the temperature-sensitive shuttle vector pBASE6 (Geiger et al., 2012). A chromosomal region encompassing *atpA* was PCR amplified from WT *S. aureus* JE2 chromosomal DNA using primer pair: 5′-ATATGAGCTCGAAGAGTTAGATAAGATTGTCAAACTAG-3′/5′-GATACAAGATCTGATGGTTTGTATTGCTACTTGC-3′ and cloned into pBASE6 *via* SacI/BglII. This plasmid was purified from *E. coli* IM08B (Monk et al., 2015) and transformed directly into JE2 *atpA*::ΦNΣ (NE592) at 30°C followed by chromosomal integration by plating on TSA (10 μg/ml chloramphenicol) at 44°C overnight. Plasmid cross-out was performed by passage at 30°C followed by plating on TSA (500 ng/ml anhydrotetracycline) and successful allelic exchange of the transposon insertion with the intact *atpA* gene was selected for by replica plating of colonies and screening for sensitivity toward erythromycin and chloramphenicol. Reconstruction of the *atpA* locus was verified by PCR amplification using primers 5′-CAAGTATGCTAAAGCATTATTTGACGTGTC-3′/5′-CGTAATTTCTGCTTGTCTCGCTCTG-3′ positioned outside the chromosomal region used for homologous recombination.

## Results and Discussion

### Screening for Hyper-Susceptibility to Antimicrobial Agents

**FIGURE 1:**
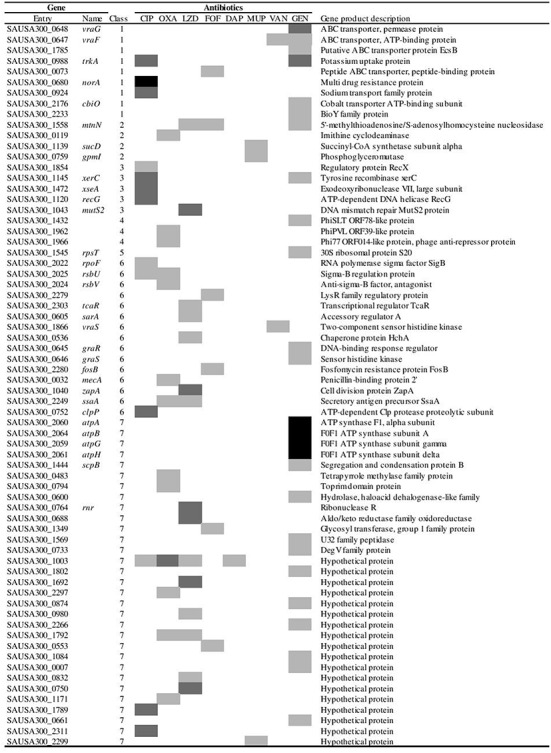
**Gene inactivations affecting susceptibility to antimicrobial agents to one or more of the eight antimicrobial agents tested.** The effect on susceptibility is displayed using a gray scale indicating fold reductions in sensitivity: 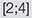, 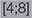 and 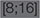. The genes are divided into the categories: (1) Membrane transporters, (2) Metabolism, (3) Replication and DNA repair, (4) Pro-phage encoded genes, (5) Protein synthesis, (6) Transcriptional regulators, chaperones, cell wall and membrane stress mechanisms and (7) Unassigned and hypothetical genes. CIP (Ciprofloxacin), OXA (Oxacillin), LZD (Linezolid), FOF (Fosfomycin), DAP (Daptomycin), MUP (Mupirocin), VAN (Vancomycin) and GEN (Gentamicin).

The complete NTML of 1920 single-gene inactivations (Fey et al., 2013) was screened for mutants displaying increased susceptibility to eight antimicrobial agents with different mechanism of action, by inspecting for lack of growth on agar plates supplemented with 0.5x MIC of the respective antimicrobials. The antimicrobials tested were ciprofloxacin, oxacillin, linezolid, fosfomycin, daptomycin, mupirocin, vancomycin, and gentamicin (**Table 1**). For the mutants identified in the initial screen as being unable to grow at 0.5x MIC, the MIC was determined using *E*-tests. A total of 68 mutants were confirmed to display twofold or greater increase in susceptibility to one or more antimicrobial agents compared to the WT strain *S. aureus* JE2 (**Figure 1**). Eight of these strains displayed increased sensitivity to multiple agents.

**Table 1:** Antimicrobial agents used in the screening for intrinsic resistance determinants, as well as the primary target and process affected by the respective agents.

Antimicrobial	Primary target	Process affected	JE2 MIC (μg/ml)
Ciprofloxacin	DNA gyrase	DNA replication	32
Oxacillin	Transpeptidase	Cell wall synthesis	0.5
Linezolid	50S rRNA subunit	Protein synthesis	3
Fosfomycin	MurA	Cell wall synthesis	0.75
Daptomycin	Cytoplasmic membrane	Membrane permeability	0.19
Mupirocin	Isoleucyl t-RNA	Protein synthesis	0.125
Vancomycin	NAM/NAG peptides	Cell wall synthesis	1.5
Gentamicin	30S rRNA subunit	Protein synthesis	1.5

*The MIC (μg/ml) of wild type strain JE2 is displayed for each agent*.

### Inactivation of Acquired Resistance Genes Increase Susceptibility to Oxacillin and Fosfomycin

The *S. aureus* JE2 strain carries the acquired resistance genes *mecA* and *fosB* on the chromosome (Fey et al., 2013), which confer resistance to β-lactams (Zapun et al., 2008) and fosfomycin (Thompson et al., 2014), respectively. As a verification of the screen, we indeed identified the *mecA* and *fosB* mutants as more susceptible to oxacillin and fosfomycin, respectively (**Figure 1**). In our experimental setup the MIC values of oxacillin and fosfomycin for WT were within the susceptibility range according to CLSI (CLSI, 2009), even though they possess *mecA* and *fosB*. Choice of medium and NaCl concentration may affect the absolute MIC value for oxacillin (Huang et al., 1993) and discrepancies in absolute fosfomycin MIC between E-test and liquid assays has been reported (Díez-Aguilar et al., 2013; Mihailescu et al., 2014). Additionally, *S. aureus* JE2 is resistant to ciprofloxacin due to mutations in the essential topoisomerase genes *gyrA* and *grlA* (Sreedharan et al., 1990; Ferrero et al., 1995; Diep et al., 2006; Highlander et al., 2007; Kennedy et al., 2008). The lowering in MIC for these three antimicrobials is potentially related to the effect of the resistance genes or resistance mutations and may not necessarily classify as intrinsic resistance determinants. *S. aureus* JE2 is susceptible toward the remaining antimicrobials and the inactivations of genes conferring increased susceptibility are considered intrinsic resistance determinants.

### Gentamicin

Gentamicin belongs to the bactericidal antibiotic class of aminoglycosides, which interferes with protein synthesis through binding to the 16S rRNA of the 30S small ribosomal unit causing mistranslation of proteins (Shakil et al., 2008). Aminoglycosides are polycationic compounds, which is an important feature for their cell interactions and uptake (Shakil et al., 2008). Twenty six mutants displayed increased sensitivity to gentamicin (**Figure 1**). Inactivation of the gene *rpsT* encoding 30S ribosomal protein S20 was found to render the cell more susceptible to gentamicin suggesting that alteration of the ribosome assembly can influence gentamicin sensitivity through some currently unknown mechanism.

Six mutants with annotated membrane transport proteins exhibited increased sensitivity to gentamicin, with the most profound effect on sensitivity observed for *vraG* and *trkA*, displaying sixfold reductions in MIC. Gentamicin uptake into the cell is known to be dependent on membrane potential, where hyperpolarization of the membrane leads to increased uptake, while depolarization reduces the uptake of gentamicin (Taber et al., 1987). Hyperpolarization and accordingly increased gentamicin uptake is suspected for the *trkA* mutant, as a *trkA* deletion mutant in *Mycobacterium smegmatis* displays hyperpolarization and increased susceptibility to aminoglycosides (Castañeda-García et al., 2011). Lack of the TrkA transporter also potentiates the efficacy of aminoglycosides in *Pseudomonas aeruginosa* (Lee et al., 2009; Gallagher et al., 2011). Inactivation of *vraG* has previously been shown to increase the susceptibility toward gentamicin, vancomycin and cationic antimicrobial peptides in *S. aureus*, potentially by alteration of the cell surface charge (Meehl et al., 2007; Yang et al., 2012). The greatest sensitization for gentamicin, being a 16-fold reduction in MIC, was observed upon inactivation of genes encoding for subunits of the ATP synthase, namely *atpA*, *atpB*, *atpG* and *atpH*. Transduction of *atpA* into WT JE2, confirmed that transposon inactivation of *atpA* increases sensitivity of *S. aureus* toward gentamicin. Inactivation of *atpA* also potentiated efficacy of gentamicin in *E. coli*, which was hypothesized to occur *via* increased uptake of aminoglycosides due to elevated membrane potentials arising from altered respiration (Lobritz et al., 2015). While we were unable to complement the *atpA*-inactivated mutant with a functional *atpA* gene on a plasmid, we successfully performed allelic exchange of the transposon insertion with the intact *atpA* gene, generating a strain displaying gentamicin sensitivity as WT (1.5 μg/ml).

Besides the numerous established genes modulating gentamicin sensitivity, the NTML screen identified several novel genes of unknown function contributing to the intrinsic sensitivity to gentamicin (**Figure 1**).

### Ciprofloxacin

Ciprofloxacin is a fluoroquinolone interfering with bacterial replication *via* inhibition of the enzymes DNA gyrase and topoisomerase IV (Blondeau, 2004). DNA gyrase, composed of two subunits GyrA and GyrB, is essential for replication initiation as the enzyme introduces negative supercoils ahead of the replication fork (Drlica and Zhao, 1997), while topoisomerase IV, composed of GrlA and GrlB, is responsible for decatenation of the replicated daughter chromosomes (Drlica and Zhao, 1997). Interaction of ciprofloxacin with these enzymes leads to a halt in replication and ultimately to DNA strands breakage (Drlica and Zhao, 1997; Blondeau, 2004). *S. aureus* JE2 is resistant to ciprofloxacin, due to amino acid changes in the DNA gyrase GyrA (Ser84Leu) and topoisomerase IV GrlA (Ser80Tyr), encoded by *gyrA* and *grlA* respectively (Sreedharan et al., 1990; Ferrero et al., 1995).

Elucidation of the intrinsic resistome in *E. coli* revealed that multiple gene products related to DNA replication and repair aid this bacterium to survive DNA damage generated by fluoroquinolone treatment (Tamae et al., 2008; Liu et al., 2010). Four of the 13 identified mutants in our screen with increased sensitivity to ciprofloxacin also display inactivation of genes related to DNA replication and repair, namely *recG*, *xerC*, *recX* and *xseA* (**Figure 1**). The RecG protein is a DNA helicase and strains with deletion of *recG* display reduced recombination and DNA repair (Al-Deib et al., 1996). Identification of *recG* corroborates previous work in *S. aureus* on increased susceptibility to quinolones in a *recG* mutant (Niga et al., 1997). A mutant of *xseA*, encoding the large subunit of exonuclease VII, was also observed in *E. coli* to be hypersensitive to ciprofloxacin (Tamae et al., 2008).

Three mutants with inactivated membrane transport systems, *norA*, *trkA* and *SAUSA300_0924*, are at least fourfold more sensitive to ciprofloxacin than the WT strain. This might be due to decreased efflux of this agent, as ciprofloxacin has been observed to being subjected to cellular efflux by pump activity of multiple efflux systems, e.g., NorA (Yamada et al., 1997; Muñoz-Bellido et al., 1999). Moreover, it was confirmed that inactivation of the stress response sigma factor σ^B^ encoded by *rpoF* and the σ^B^ activator RsbU potentiated the effect of ciprofloxacin, which is in agreement with previous observations in *S. aureus* (Riordan et al., 2006). We also identified the *clpP* gene encoding the proteolytic subunit of the ClpXP two-component protease to be more susceptible to ciprofloxacin, corroborating a study in *P. aeruginosa* (Fernández et al., 2012). A possible link between *clpP* and ciprofloxacin susceptibility is that *clpP* has been shown to interfere with activation of the SOS DNA-damage response (Cohn et al., 2011). Additionally, inactivation of several hypothetical genes (*SAUSA300_1789* and *SAUSA300_2311*) also conferred a fourfold or greater sensitization to ciprofloxacin relative to the WT strain.

### Linezolid

Linezolid belongs to the antimicrobial class of oxazolidinones (Moellering, 2003). Linezolid interacts with the A-site of the 50S ribosomal subunit and inhibits formation of the initiation complex, thus inhibiting protein synthesis (Wilson, 2014). A total of 15 mutants were more susceptible to linezolid (**Figure 1**), with six mutants displaying at least a fourfold increased sensitivity. None of the identified genes have to our knowledge previously been associated with increased linezolid susceptibility. Interestingly, these genes exert widely different functions, such as *zapA* encoding the divisome protein ZapA, which stabilizes Z-ring formation (Adams and Errington, 2009), *mutS2* encoding MutS2 that in *Helicobacter pylori* function as an inhibitor of recombination (Pinto et al., 2005) and the ribonuclease R.

### Mupirocin

The target of mupirocin is the isoleucyl-tRNA synthetase (IleRS), thus inhibiting aminoacylation of isoleucine to the cognate tRNA and thereby prevents protein synthesis (Yanagisawa et al., 1994; Pope et al., 1998). Only three mutants exhibited increased sensitivity toward mupirocin (**Figure 1**). None of the mutants, *sucD*, *gpmI* and *SAUSA300_2299*, have previously been associated with altered susceptibility to mupirocin. The gene *SAUSA300_2299* has been annotated as a putative multidrug resistance transporter and could be involved in active efflux of mupirocin from the cytoplasm.

### Oxacillin

Oxacillin is a β-lactam interfering with cell wall biosynthesis. The bactericidal activity of oxacillin derives from its binding to penicillin-binding proteins (PBPs), thus preventing cross-linking of peptidoglycan units of the cell wall, which eventually causes cell lysis (Kotra and Mobashery, 1998). In our study, we identified 13 genes displaying increased susceptibility to oxacillin (**Figure 1**). *S. aureus* JE2 contains the *mecA* gene encoding the alternative penicillin binding protein 2a (PBP2a) that enables cross-linking of peptidoglycan units in the presence of β-lactams (Zapun et al., 2008). Indeed inactivation of *mecA* rendered the strain more susceptible to oxacillin. A functional σ^B^ response is important for oxacillin susceptibility (Singh et al., 2003; Schulthess et al., 2009) and RsbU together with RsbV are activators of this stress response (Palma and Cheung, 2001), both of these determinants were confirmed as targets for oxacillin sensitizers in our screen. In addition to the previously established contributors to oxacillin intrinsic resistance, several genes of unknown function were identified (*SAUSA300_1792* and *SAUSA300_2297*) that upon inactivation increased susceptibility to oxacillin equally well as *mecA* inactivation. It was also observed that inactivation of the unknown gene *SAUSA300_1003* displayed even greater sensitivity to oxacillin than the *mecA* mutant. Moreover, this *SAUSA300_1003* mutant displayed increased sensitivity to ciprofloxacin, linezolid and daptomycin and thereby it could be of interest to investigate how this gene can potentiate the efficacy of multiple antimicrobial agents.

### Fosfomycin

Fosfomycin is a phosphoenolpyruvate analog that inhibits the first enzymatic step in peptidoglycan biosynthesis, by binding to the enzyme MurA and thereby prevent the formation of *N*-acetylmuramic acid, an essential precursor of the peptidoglycan cell wall (Michalopoulos et al., 2011; Karageorgopoulos et al., 2012). *S. aureus* JE2 harbors the fosfomycin resistance gene (*fosB*) that chemically inactivates fosfomycin (Rigsby et al., 2005; Castañeda-García et al., 2013) and in agreement, the *fosB* mutant showed increased sensitivity to fosfomycin. Moreover, inactivation of the gene *SAUSA300_2279* encoding a regulatory protein of the LysR family also increased susceptibility to fosfomycin. This gene is located directly upstream of *fosB* and could potentially be a positive regulator of *fosB* or the transposon insertion in *SAUSA300_2279* could prevent proper transcription of *fosB*. Four other genes also affected fosfomycin susceptibility, with a hypothetical (*SAUSA300_0553*) and a putative peptide transporter (*SAUSA300_0073*) being most prominent with threefold greater sensitivity to fosfomycin, which equal the potentiating effect of the *fosB* mutant.

### Vancomycin

Vancomycin is a glycopeptide antibiotic readily used in the treatment of methicillin-resistant *S. aureus* (Liu et al., 2011). The bactericidal activity of vancomycin is derived from the inhibition of cell wall biosynthesis. Vancomycin binds to the D-Ala-D-Ala residues of the pentapeptides on the peptidoglycan precursors *N*-acetylmuramic acid and *N*-acetylglucosamine and thus prevents peptide cross-linking of the polysaccharide backbone in peptidoglycan biosynthesis (Reynolds, 1989; Boger, 2001). Inactivation of two genes increased the susceptibility to vancomycin, with a maximum two-fold reduction in MIC (**Figure 1**). We confirmed the previously established vancomycin intrinsic resistance determinants *vraS* (Kuroda et al., 2003; Gardete et al., 2012) and *vraF* of the VraFG ABC transporter system (Meehl et al., 2007). Although our screen identified recognized vancomycin intrinsic resistance determinants, we did not observe mutants of the *dlt* operon as seen previously in a corresponding vancomycin hypersusceptibility screen (Blake and O’Neill, 2013). No strains in the NTML exist with inactivation of any of the four genes in the *dlt* operon, which is involved in adding positively charged D-alanine to teichoic acids (Peschel et al., 1999).

### Daptomycin

Daptomycin is a cyclic lipopeptide displaying bactericidal activity against many Gram-positive organisms (Steenbergen et al., 2005). The bactericidal activity of daptomycin is attributed to the insertion of the lipophilic tail into the bacterial cell membrane, leading to rapid membrane depolarization and potassium ion efflux, with downstream arrest of DNA, RNA and protein synthesis (Steenbergen et al., 2005).

Only a single mutant, *SAUSA300_1003*, displayed increased susceptibility to daptomycin. The gene *SAUSA300_1003* encodes a protein of unknown function and this mutant is also more susceptible to several of the other antimicrobial agents tested.

### Potentiating the Effect of Gentamicin in the *Galleria mellonella* Infection Model

**FIGURE 2:**
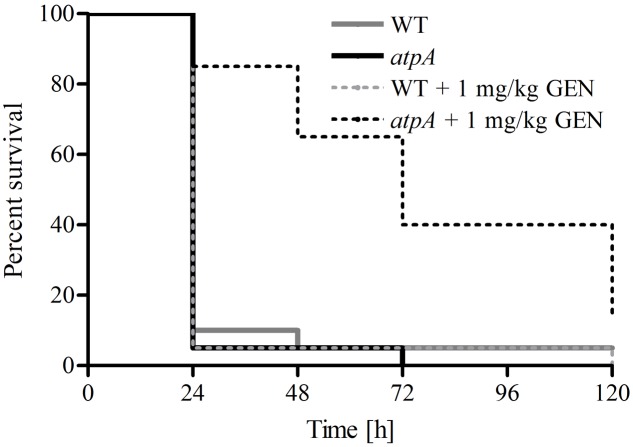
**Effect of gentamicin treatment of *G. mellonella* larvae inoculated with 1 × 10^7^ CFU of *S. aureus* JE2 and the *atpA* mutant.** Gentamicin (1 mg/kg bodyweight) were administered 1 h post infection with follow-up treatments at 24, 48, and 72 h. Prolonged survival of *G. mellonella* inoculated with *atpA* due to gentamicin treatment was observed. All larvae survived treatment with only gentamicin (1 mg/kg bodyweight).

To verify the potential of enhancing the efficacy of antimicrobial agents by targeting a non-essential gene product in *S. aureus*, we employed the *G. mellonella* infection model (Ramarao et al., 2012). We tested the efficacy of gentamicin at a clinically relevant dose (1 mg/kg bodyweight) to promote survival of *G. mellonella* infected with a lethal dose (10^7^ cells/larvae) of *S. aureus* JE2 (WT) and the *atpA* mutant (**Figure 2**). Gentamicin significantly prolonged survival of *G. mellonella* infected with the *atpA* mutant compared to the treatment of WT (*P* < 0.0001). None of *G. mellonella* larvae in the group only receiving gentamicin treatment died during the experiment (Data not shown).

## Conclusion

In this work, we have identified genetic determinants that increase the sensitivity of *S. aureus* to eight antimicrobial agents. The many modulators of antimicrobial susceptibility uncovered here for *S. aureus* JE2 comprise both previously established determinants in addition to numerous novel genes. To our knowledge this study provides the first whole genome overview of intrinsic resistance genes of *S. aureus* to agents with different modes of action. With the results obtained, it will be feasible to select particular genes for further investigation as targets for antimicrobial potentiators. The mechanisms by which the majority of these determinants contribute to modulate antibiotic susceptibility remain unknown and further work is required to establish this.

It is important to note that *S. aureus* JE2 carries resistance determinants to oxacillin and fosfomycin as well as mutations conferring resistance to ciprofloxacin and the genes identified for these agents cannot *per se* be classified as intrinsic resistance genes, unless gene inactivation is examined in a susceptible strain. However, the gene inactivations identified here to reduce the MIC of these agents could potentially be explored for re-sensitizing resistant *S. aureus* isolates.

*Staphylococcus aureus* JE2 was not phenotypically resistant to the remaining antibiotics tested (linezolid, vancomycin, gentamicin, daptomycin and mupirocin). However, an antimicrobial agent such as gentamicin has limited use against *S. aureus* due to the risk of toxicity at the required clinical concentrations (Rayner and Munckhof, 2005). Therefore, based on our results co-administration of an inhibitor targeting the ATP synthase could potentially lower the concentration of gentamicin needed for treatment of *S. aureus* and thus increase the usefulness of this drug. In the *G. mellonella* infection model we observed significantly increased survival of larvae infected with *atpA* compared to WT, when treated with gentamicin at similar concentrations used for human therapy (Rayner and Munckhof, 2005).

Improved activity of vancomycin could also be of clinical significance, even for strains that are already susceptible to this agent. There is an inverted association between MIC and treatment efficiency of vancomycin against methicillin-resistant *S. aureus* infections, even with MIC values well within the susceptibility range (Sakoulas et al., 2004; Lodise et al., 2008). A further reduction of the vancomycin MIC with a helper-drug could thus increase the probability of vancomycin treatment success.

Taken together, our observations provide a framework for understanding the contribution of chromosomal determinants that affect the susceptibility to antimicrobial agents in *S. aureus* and provide novel targets for further exploration in development of small molecules to be used as antimicrobial potentiators.

## Author Contributions

MV and HI conceived and designed the study. Experiments were performed by MV, BL, MB, and JH. All authors contributed in analysis of data and drafting the manuscript. All authors read and approved the final manuscript.

## Conflict of Interest Statement

The authors declare that the research was conducted in the absence of any commercial or financial relationships that could be construed as a potential conflict of interest.
